# Supplementary material for: Myoglobin–Pyruvate Interactions: Binding Thermodynamics, Structure–Function Relationships, and Impact on Oxygen Release Kinetics
Source: Int J Mol Sci. 2022 Aug 6;23(15):8766. doi: 10.3390/ijms23158766 (PMC9369265; doi:10.3390/ijms23158766)

Myoglobin-Pyruvate Interactions: Thermodynamic Binding, Structure-Function Relationships, and Impact on Oxygen Kinetics

Kiran Kumar Adepu [1,2,*,†], Dipendra Bhandari [1,†], Andriy Anishkin [3], Sean H. Adams [4,5] and Sree V. Chintapalli 1,2,*

Figure S1. Effect of PYR binding to oxy-Mb and O_2 release. Representative graph showing O_2 release from oxy-Mb after addition of 5 mM PYR at pH 6.4 and pH 6.0.

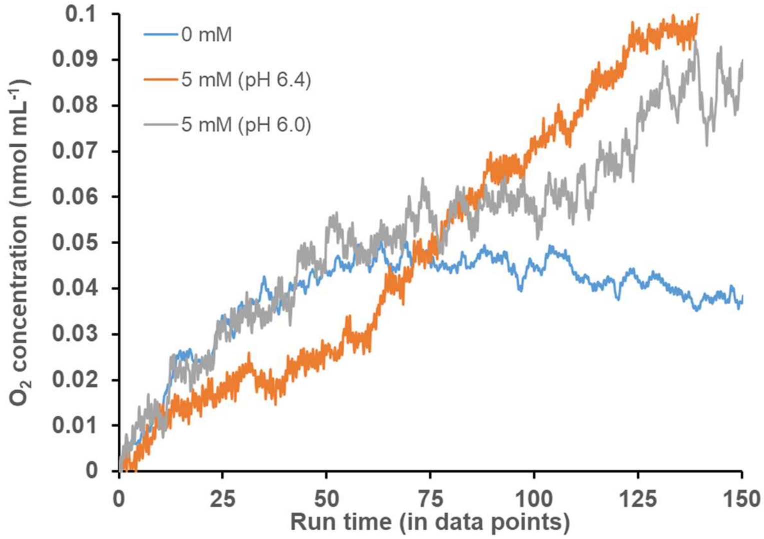

Figure S2. Autodock results displaying LAC interaction with oxy-Mb residues at (**a**) pH 7.0, (**b**) pH 6.4, and (**c**) pH 6.0, and (d) deoxy-Mb. With oxy-Mb, at pH 7.0, LAC shows interaction with residues K45, K63, and D60, while at pH 6.4, LAC shows interaction with residues K41, H96, and K97, and at pH 6.0, LAC shows interaction with residues K56 and E59. While with deoxy-Mb, irrespective of the change in acidic pH (pH 6.4 and pH 6.0), LAC is docked near to proximal His side of heme center of deoxy-Mb, interacting with the residues H96 and S92, except at pH 7.0, where no LAC binding was observed. LAC (brown), heme center (pink), and the residues (green) interacting with LAC are displayed as sticks. Mb protein (cyan) is displayed as ribbon structure an oxygen (red) in spheres. Possible hydrogen bond interactions between side chains of residues and LAC are displayed as dashed yellow lines with bond length.

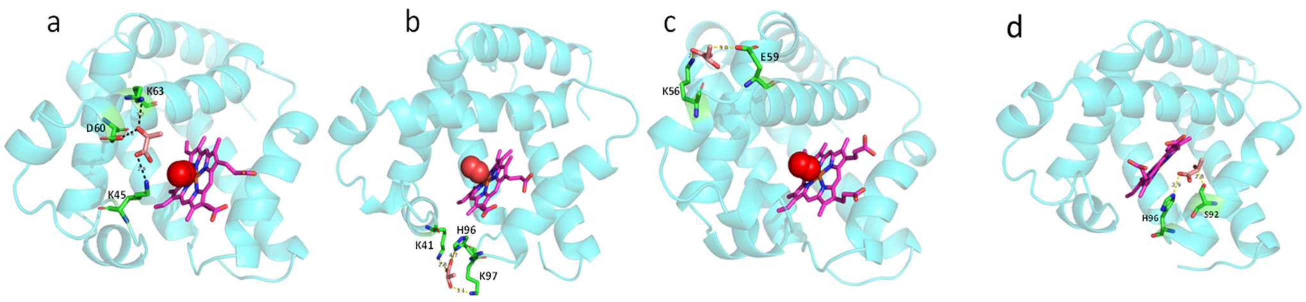

Figure S3. Representative ITC plots of binding of PYR with LYZ at (**a**) pH 7.0 (**b**) pH 6.4 and (**c**) pH 6.0.

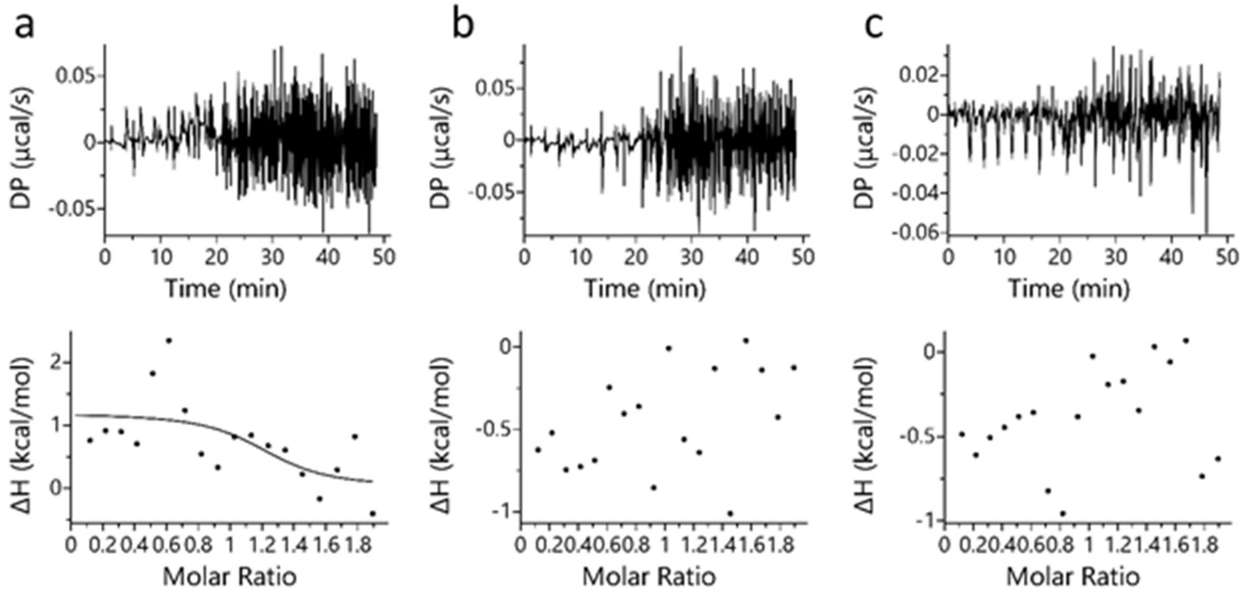

Figure S4. Representative CD spectra of secondary structural analysis of Mb with varying concentration of PYR. Top row displays oxy-Mb and bottom row displays deoxy-Mb at (**a**,**d**) pH 7.0, (**b**,**e**) pH 6.4, and (**c**,**f**) pH 6.0.

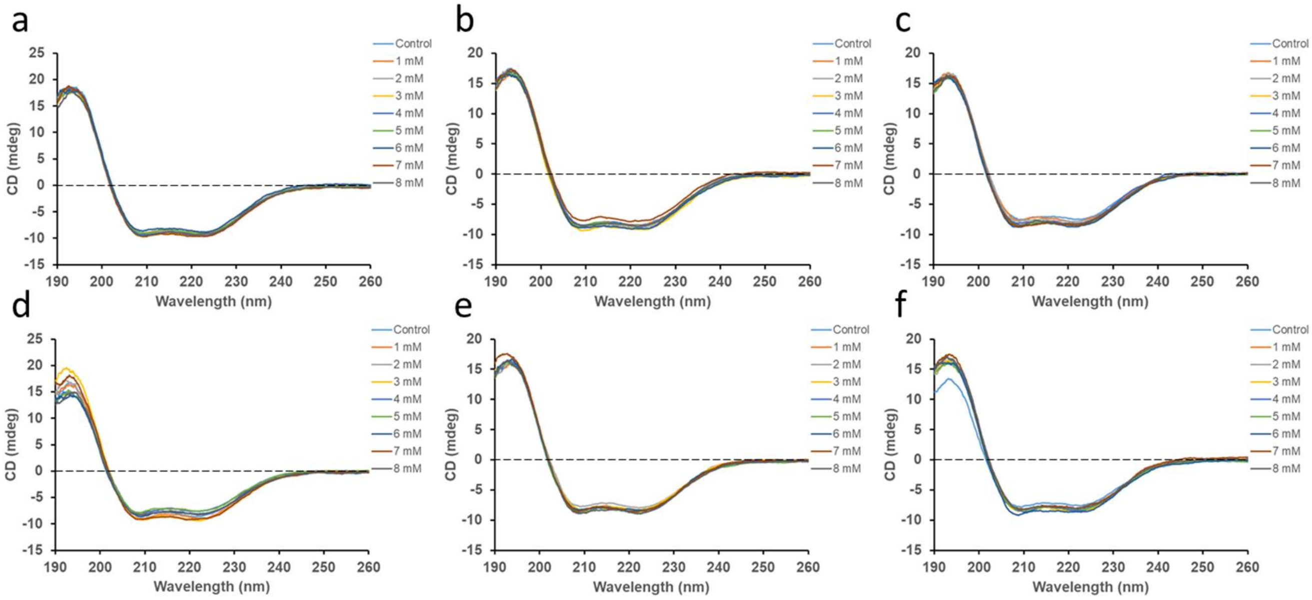

Figure S5. Representative CD spectra of secondary structural analysis of Mb with varying concentration of LAC. Top row displays oxy-Mb and bottom row displays deoxy-Mb at (**a**,**d**) pH 7.0, (**b**,**e**) pH 6.4, and (**c**,**f**) pH 6.0.

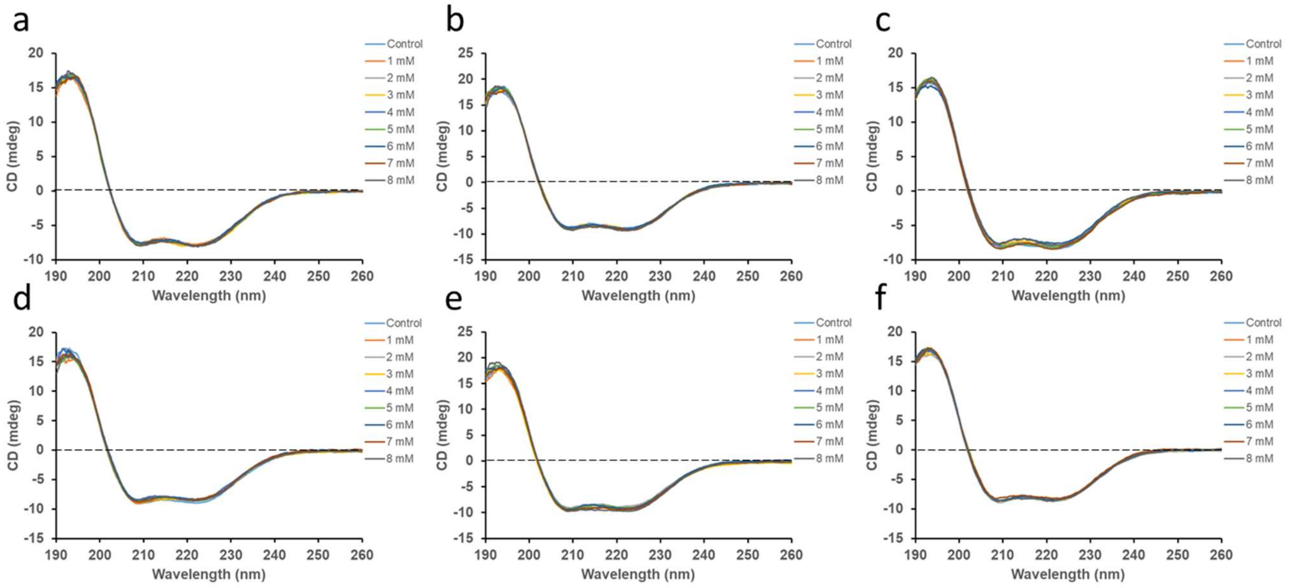

Supplement: Supplementary file 1 [file ijms-23-08766-s001.zip › ijms-1841665-supplementary.pdf]
